# Mind Matters: Treatment Concerns Predict the Emergence of Antiretroviral Therapy Side Effects in People with HIV

**DOI:** 10.1007/s10461-018-2239-6

**Published:** 2018-09-05

**Authors:** Rob Horne, Sarah Chapman, Elizabeth Glendinning, Heather Leake Date, Jordi Guitart, Vanessa Cooper

**Affiliations:** 1 Centre for Behavioural Medicine, UCL School of Pharmacy, BMA House, Tavistock Square, London, WC1H 9JP UK; 2 Departments of Pharmacy and HIV Medicine, Brighton & Sussex University Hospitals NHS Trust, Brighton, UK; 3 Spoonful of Sugar Ltd, UCL Business PLC, London, UK

**Keywords:** HIV, Adverse effects, Beliefs, Antiretroviral therapy

## Abstract

The aim of this analysis of historical data was to determine whether patients’ pre-treatment beliefs about antiretroviral therapy (ART) predict the subsequent reporting of side effects. Data were collected as part of a prospective, 12-month follow-up study. Of 120 people starting ART, 76 completed follow-up assessments and were included in the analyses. Participants completed validated questionnaires assessing their beliefs about ART, beliefs about medicines in general, perceived sensitivity to adverse effects of medicines, depression and anxiety before initiating ART and after 1 and 6 months of treatment. Adherence was assessed at 1, 6 and 12 months. Pre-treatment concerns about ART were associated with significantly more side effects at 1 month (p < 0.05) and 6 months (p < 0.005). Side effects at 6 months predicted low adherence at 12 months (p < 0.005). These findings have implications for the development of interventions to support patients initiating ART by providing a mechanism to pre-empt and reduce side effects.

## Resumen

El objetivo de este análisis de datos históricos fue determinar si las creencias de los pacientes sobre el tratamiento antirretroviral (TAR) predecían la declaración posterior de efectos secundarios. Los datos fueron recogidos como parte de un estudio prospectivo de 12 meses. De las 120 personas que comenzaron el TAR, 76 completaron las evaluaciones de seguimiento y se incluyeron en los análisis. Los participantes respondieron a cuestionarios validados que evalúan sus creencias sobre el TAR, creencias sobre los medicamentos en general, la sensibilidad percibida ante los efectos adversos de los medicamentos, la depresión y la ansiedad antes de iniciar el TAR y transcurridos 1 y 6 meses de tratamiento. La adherencia al TAR se evaluó a 1, 6 y 12 meses. Las preocupaciones previas al inicio del tratamiento acerca del TAR se asociaron con un número significativamente mayor de efectos secundarios al cabo de 1 mes (p < 0.05) y 6 meses (p < 0.005). Los efectos secundarios a los 6 meses predijeron baja adherencia a los 12 meses (p < 0.005). Los resultados de este análisis tienen implicaciones en el desarrollo de intervenciones para apoyar a los pacientes que comienzan el TAR, proporcionando un mecanismo para adelantarse y reducir los efectos secundarios.

## Introduction

Antiretroviral therapy (ART) extends life expectancy, improves quality of life and prevents transmission of HIV [1–3]. Compared with first generation ART, newer agents have been associated with a lower incidence of treatment-limiting side effects, such as liver toxicity and changes in fat distribution. However non-specific side effects such as fatigue and nausea are still frequently reported [4, 5]. Although there is often no recognised pharmacological explanation, these side effects cause distress and impair quality of life [5]. Moreover, side effects are among the most common reasons for treatment switches and nonadherence to ART [6, 7] and therefore contribute to suboptimal treatment outcomes.

Side effects are commonly defined as the action of a drug other than the one intended. However, not all side effects can be directly attributed to the pharmacological action of the drug. Many patients experience side effects when taking placebo [8], indicating that side effects can be caused by factors other than the drug itself. These negative, nonspecific effects of a drug are described as nocebo responses [9]. Factors such as verbal suggestion and negative treatment experiences can induce side effects in people taking both placebos and active drug treatment [10, 11]. A better understanding of the determinants of side effects of ART could help healthcare professionals to identify patients who are at risk of developing side effects and offer appropriate support.

Beliefs about medicines are reliable predictors of ART nonadherence and discontinuation [12–14]. Studies in other long-term conditions have found associations between patients’ beliefs about treatment and their experience of side effects. For example, patients’ pretreatment expectations of side effects from cancer treatments predicted the subsequent reporting of side effects [15, 16]. Concerns about medicines have been associated with side effects both in cross-sectional studies of Medicaid enrolees [17, 18] and in a prospective study, where heightened concerns about treatment for rheumatoid arthritis independently predicted side effects 6 months later [19]. Patients who perceived themselves to be particularly sensitive to adverse effects from medicines were more likely, than people without this belief, to report symptoms in the week following vaccination [20]. Negative affect and trait anxiety have also been found to predict side effects [16, 20].

Little is known about psychological determinants of side effects in people taking ART. A cross-sectional study found that patients’ concerns about ART were associated with a greater number of symptoms reported by patients, but due to the cross-sectional design of the study it was not possible to determine whether experiencing symptoms led to treatment concerns, or whether pre-existing concerns about ART led to a greater number of symptoms [14]. Furthermore, it was not clear whether patients attributed the symptoms to HIV, ART or another cause.

To our knowledge, no studies to date have evaluated whether specific and general medication beliefs, or other psychological factors such as depression and anxiety, predict side effect reports in HIV. We therefore set out to explore this in historical data from a prospective study conducted from 2000 to 2004 which measured patients’ beliefs about medicines, experiences of side effects, depression, anxiety and adherence to ART over the first 12 months of a new ART regimen. Since specific medication concerns are often related to more general beliefs about pharmaceuticals and perceptions of personal sensitivity to the effects of medicines (collectively known as ‘pharmaceutical schema’ [21]), we explored whether patients’ beliefs about medicines in general and perceived sensitivity to medicines were associated with concerns about ART. We believe that the use of historical data is appropriate to address this question despite advances in antiretroviral regimens and side effect profiles, because the question of whether beliefs about medicines predict the reporting of side effects is not dependent on the type of side effects reported.

## Methods

This analysis was conducted using data collected as part of a prospective study of uptake and adherence to ART among HIV positive patients attending the Lawson Unit, a National Health Service (NHS) HIV clinic in Brighton, United Kingdom (for further details see [12]). Patients completed questionnaires before initiating ART (baseline) and after 1 and 6 months. Self-reported adherence was measured at 1, 6 and 12 months.

### Participants

Patients were eligible for the study if they were HIV positive, attending the Lawson Unit, and not taking antiretroviral therapy. Participants who subsequently accepted a clinically indicated offer of ART formed the study sample and were followed up for a year. Exclusion criteria were having insufficient understanding of English or being too ill to complete the study questionnaires.

### Procedure

Consecutive study-eligible individuals were referred to the study by their HIV doctor. Standard procedures for obtaining written informed consent were followed. Researchers attended weekly multidisciplinary meetings to identify patients who were eligible for an offer of ART based on contemporaneous British HIV Association (BHIVA) treatment guidelines [22]. Following a treatment recommendation, participants were given a questionnaire to complete along with a stamped addressed envelope for its return. Medical notes and pharmacy records were consulted to identify participants who initiated ART. These participants were sent follow-up questionnaires after 1, 6 and 12 months. Telephone reminders were administered to optimise response rates.

### Measures

#### ART Side Effects

ART side effects were measured using an adapted version of the Identity subscale of the Illness Perceptions Questionnaire-Revised (IPQ-R) [23]. The original IPQ-R has been validated across multiple illness groups [23]. The IPQ-R symptoms scale comprises 11 core symptoms common to a variety of illnesses. We modified the scale for this study by adding 12 common HIV/ART related symptoms. Participants were asked to indicate which if any of the symptoms they experienced as a result of taking ART. If they had experienced the symptom they were asked to rate the severity of the symptom on a scale of 1–5, where 1 = very mild, 2 = mild, 3 = moderate, 4 = severe and 5 = very severe. To ensure that only symptoms that were problematic to the patient were included, we counted the number of symptoms that the participants rated as moderate, severe or very severe (scored 3–5). This gave a possible score of 0–23, representing the number of symptoms that the participant perceived to be moderate, severe or very severe.

#### Beliefs About Medicines

Beliefs about medicines were measured using the Beliefs about Medicines Questionnaire (BMQ) [24, 25]. We used both General and Specific versions—the BMQ-General, assesses beliefs about medicines as a whole, and the BMQ-Specific, assesses beliefs about a specific prescribed medicine. The BMQ-General compares three subscales, each consisting of 4 items. The General Harm scale assesses beliefs that medicines are harmful, addictive poisons; the General Overuse scale assesses beliefs that medicines are overused by doctors, and General Benefits scale assesses beliefs that medicines generally are beneficial. Scores for individual items within each scale were summed. A mean score was computed by dividing each total score by the number of items, giving a range of 1–5 for each scale. Beliefs about ART were measured using the BMQ-Specific-ART specific version (BMQ-ART) [12, 24] which has previously been validated in people living with HIV [26]. The BMQ-ART comprises two scales: an ART-Necessity scale consisting of 6 items assessing participants’ perceptions of their personal need for ART for controlling HIV and maintaining health, and an ART-Concerns scale, consisting of 7 items assessing concerns about potential adverse consequences of ART (e.g. concerns about short and long-term effects and the concern that taking ART would disrupt daily life). Participants were presented with a series of statements and asked to rate their level of agreement with each on a scale where possible responses ranged from strongly agree to strongly disagree. After reverse scoring relevant items, scores for individual items within each scale were summed. A mean score was computed by dividing each total score by the number of items, giving a range of 1–5 for both Necessity and Concern scales, with higher scores representing stronger necessity beliefs and concerns.

#### Perceived Sensitivity to Medicines (PSM) Scale

Participants rated 5 items concerning their body’s response to medicines on a 5-point Likert scale anchored from ‘strongly agree’ to ‘strongly disagree’ [27]. Individual item scores were summed to provide a total PSM score ranging from 5 to 25. In order to facilitate comparison of scores between scales, a mean score was computed by dividing by the number of items, giving a range of 1–5. The PSM scale has been shown to have adequate reliability and validity [27].

#### Hospital Anxiety and Depression Scale (HADS)

The HADS is a brief measure of state anxiety (7 items) and depression (7 items) which was developed to determine clinical anxiety/depression and determine the severity of symptoms in patients attending outpatient clinics, without contamination of scores by reporting of physical symptoms. There was no overlap between the items on the HADS and the items included on our measure of ART side effects. Items were scored from 0 to 3 according to a scoring manual. Possible scores range from 0 to 21. Higher scores indicate greater anxiety or depression. Scores of 8–10 in each subscale indicate possible clinical disorder, scores of 11–21 indicate probable clinical disorder. The HADS has demonstrated good psychometric properties in studies of medical outpatients [28].

#### Adherence

Adherence to ART was measured using a visual analogue scale (VAS) from the Medication Adherence Self-Report Inventory (MASRI) [29]. The MASRI is worded in a nonjudgmental manner in an effort to minimize socially desirable responses. The VAS has demonstrated good validity against objective measures (electronic monitoring: r = 0.63, p < 0.001; pill count: r = 0.75, p < 0.001; and viral load: p < 0.01) [29]. Participants were asked to estimate on a visual analogue scale from 0 to 100 the percentage of medication they had taken as prescribed over the previous month. Those who reported taking less than 95% of ART medicines were categorised into a low adherence group, and those taking 95% or more of their medication as prescribed were allocated to a high adherence group. The 95% threshold was chosen on the basis of research linking ≥ 95% adherence to undetectable viral load [30].

#### Clinical and Demographic Data

Clinical and demographic information, including age, sex, employment status, sexual orientation, number of years since first HIV diagnosis, symptom classification (asymptomatic HIV, symptomatic HIV or AIDS), whether the person had previously been prescribed ART, CD4 count and viral load (log_10_) was extracted from participants’ medical records.

#### Analysis

Data were analysed using IBM SPSS Statistics 24. Clinical and demographic characteristics of the sample and questionnaire scores at baseline were compared between those who completed the study and those with missing data at 1 and 6 months using χ^2^ tests for categorical data and independent samples t-tests for continuous variables. Cochran’s Q test was used to compare the number of participants reporting low adherence across 1, 6 and 12 months. Pearson’s correlations were used to assess associations between clinical, demographic and psychological variables and (1) the number of moderate, severe or very severe side effects reported at 1 month and 6 months, and (2) baseline BMQ-Concerns scores. Point biserial correlations were reported where one variable was categorical. The BMQ-Concerns score was dichotomised into high concerns and low concerns by splitting the group on the median score.

In order to test the hypothesis that depression and anxiety mediate the relationship between concerns about ART and the subsequent reporting of side effects, we conducted mediational analyses in line with the recommendations of Kenny [31, 32]. We hypothesised that ART concerns at baseline would be associated with anxiety and depression at 1 month, and that anxiety and depression at 1 month would mediate the relationship between baseline concerns and side effects at 6 months. This hypothesis was tested by using multilinear regression analyses to assess relationships between the independent variable (ART-Concerns at baseline), the outcome (side effects at 6 months) and the proposed mediators (depression or anxiety at 1 month). Multilinear regression analyses were used to control for the proposed mediators (depression and anxiety) when assessing the relationship between ART-Concerns and side effects.

## Results

Four hundred and ninety-one patients were eligible for the study, and 365 (74.3%) were referred by their physician. Three hundred and twenty-two (88.2%) of those referred agreed to take part. After recruitment, 153 participants were recommended HAART and 136 (88.9%) returned questionnaires and were included in the original analysis [12]. Of those, 98 participants initially initiated treatment [12]. A further 22 participants enrolled in the study initially declined a treatment recommendation but initiated treatment over the 1 year follow-up. A total of 120 participants were therefore eligible for the current study. Over the follow-up, 17 participants stopped treatment, 3 died, and 24 were lost to follow-up, missed one or more follow-up or returned questionnaires with missing data. Seventy-six participants provided data at each time point and formed the sample for this study. Clinical and demographic characteristics of the sample are shown in Table 1.

**Table 1:** Sample demographics and clinical characteristics

Baseline clinical/demographic feature		Completed study n = 76	Missing data n = 44		p
Age (years)	Mean (SD)	40.2 (8.8)	34.2 (6.2)	t = − 4.00	< 0.001
Men who have sex with men (MSM)	n (%)	70 (92.1)	38 (86.4)	χ^2^ = 0.312	0.312
White British	n (%)	23 (30.3)	21 (47.7)	χ^2^ = 3.660	0.056
Employed	n (%)	53 (69.7)	24 (54.5)	χ^2^ = 2.797	0.094
Years since HIV diagnosis	Mean (SD)	4.6 (5.1)	3.9 (4.2)	t = 0.442	0.442
Asymptomatic HIV	n (%)	24 (31.6)	13 (29.5)	χ^2^ = 0.054	0.816
Symptomatic HIV	n (%)	34 (44.7)	17 (38.6)	χ^2^ = 0.424	0.515
AIDS	n (%)	18 (23.7)	14 (31.8)	χ^2^ = 0.943	0.332
Prior antiretroviral therapy	n (%)	20 (26.3)	24 (54.5)	χ^2^ = 9.563	0.002
CD4 count (mm^3^/L)	Mean (SD)	198.3 (119.3)	190.1 (145.2)	t = 0.736	0.736
Viral load (log_10_ copies/mL)	Mean (SD)	5.3 (0.5)	5.3 (0.5)	t = 0.953	0.605
BMQ-necessity	Mean (SD)	4.0 (0.5)	3.7 (0.6)	t = − 2.959	0.004
BMQ-concerns	Mean (SD)	3.0 (0.6)	3.2 (0.6)	t = 1.803	0.074
BMQ—general overuse	Mean (SD)	3.0 (0.7)	3.1 (0.7)	t = 0.795	0.428
BMQ—general harm	Mean (SD)	2.4 (0.5)	2.7 (0.5)	t = 2.652	0.009
BMQ—general benefits	Mean (SD)	3.9 (0.5)	3.7 (0.4)	t = − 1.689	0.095
PSM	Mean (SD)	2.5 (0.7)	3.2 (1.1)	t = 3.394	0.001
HADS depression	Mean (SD)	5.3 (4.4)	7.2 (4.6)	t = 2.192	0.030
HADS anxiety	Mean (SD)	7.5 (4.9)	10.6 (4.9)	t = 3.351	0.001

*BMQ* beliefs about medicines in general, *HADS* hospital anxiety and depression scale, *PSM* perceived sensitivity to medicines scale

### ART Side-Effects

At 1 month, 55 (72.4%) participants reported ≥ 1 moderate to severe symptom that they attributed to ART. The number of symptoms reported ranged from 0 to 17 (mean = 3.8, SD = 4.4). At 6 months, 45 (59.2%) participants reported ≥ 1 moderate to severe symptom that they attributed to ART. The number of symptoms reported ranged from 0 to 17 (mean = 3.2 SD = 4.4). The ten most common side effects reported at 6 months were sleep difficulties (29%), sexual problems (26%), fatigue (24%), skin problems (22%), diarrhoea (21%), altered sensation in hands or feet (18%), stiff joints (16%), nausea (16%), headaches (16%) and pain (15%).

### Beliefs About Medicines

Mean scores and standard deviations for scales measuring beliefs about treatment at baseline are shown in Table 2. Table 2 also shows the percentage of participants scoring above the midpoint for each scale. This provides an estimate of the proportion of participants who hold strong views about the construct measured by each scale. The vast majority (99%) of participants had strong views about the necessity of their treatment, however half the sample (51%) had strong concerns about potential adverse consequences of taking ART. Examination of individual items on the BMQ ART-Concerns scale showed that the most frequently endorsed concerns were worries about long-term effects and concerns about side effects. 58% of participants agreed or strongly agreed with the item “I would worry about the long-term effects of this medicine” while 49% agreed or strongly agreed with the item “These medicines would give me unpleasant side effects.” Most participants had fairly positive beliefs about medicines in general, with 97% scoring above the scale mid-point on the BMQ-General Benefit scale and only 15% scoring above the midpoint on the BMQ-General Harm scale, however 53% scored above the mid-point on the BMQ General Overuse scale. Almost a third of participants (32%) perceived themselves to be particularly sensitive to medicines in general as indicated by their responses to the PSM scale.

**Table 2:** Scale descriptives at the baseline assessment

Scale	Number of items in scale	Internal consistency (alpha)	Mean	Standard deviation (SD)	Percentage scoring above scale mid-point
BMQ ART-Necessity	6	0.839	4.0	0.5	98.7
BMQ ART-Concerns	7	0.764	3.0	0.6	51.3
BMQ ART-General Harm	3	0.420	2.4	0.5	14.5
BMQ ART-General Overuse	4	0.660	3.0	0.6	53.2
BMQ ART-General Benefits	4	0.645	3.9	0.5	96.7
PSM	5	0.836	2.5	0.7	32.2

*BMQ* beliefs about medicines questionnaire, *PSM* perceived sensitivity to medicines scale

### Anxiety and Depression

Scores on the HADS depression scale at baseline ranged from 0 to 19. The mean score was 5.3 (SD = 4.4). Fifty-one participants (67.1%) had scores in the range of 0–7, indicating the absence of clinically significant depression. A further 17 (22.4%) participants had scores of 8–10 indicating possible clinical depression and 8 (10.5%) had scores of 11 and above indicating probable clinical depression. Scores on the HADS anxiety scale at baseline ranged from 0 to 19. The mean score was 7.5 (SD = 4.8). Thirty-nine participants (51.3%) had scores in the range of 0–7, indicating the absence of clinically significant anxiety. A further 14 participants (18.4%) had scores of 8–10 indicating possible clinical anxiety and 23 (30.3%) had scores of 11–21 indicating probable clinical anxiety.

### Adherence to ART

The number of participants reporting < 95% adherence increased from 5 (6.6%) at 1 month to 17 (22.4%) at 12 months (Cochran’s Q = 20.32, df = 3, p < 0.001). Side effects at 6 months predicted non-adherence at 12 months: Participants with high adherence at 12 months reported a mean of 2.5 (SD = 3.9) side effects at 6 months, while those who reported low adherence at 12 months reported a mean of 5.8 (SD = 5.2) side effects at 6 months (t = − 2.905; p = 0.005). Side effects at 1 month did not significantly predict low adherence at 6 months or 12 months: participants with high adherence at 6 months reported a mean of 3.65 (SD 4.34) side effects at 1 month while those who reported low adherence at 6 months reported a mean of 4.50 side effects at 1 month (t = − 0.687; p = 0.494). Participants with high adherence at 12 months reported a mean of 3.4 (SD = 4.2) side effects at 1 month, while those who reported low adherence at 12 months reported a mean of 5.5 (SD = 4.5) side effects at 1 month (t = − 1.778, p = 0.080).

### Baseline Predictors of Side Effects

**Fig. 1:**
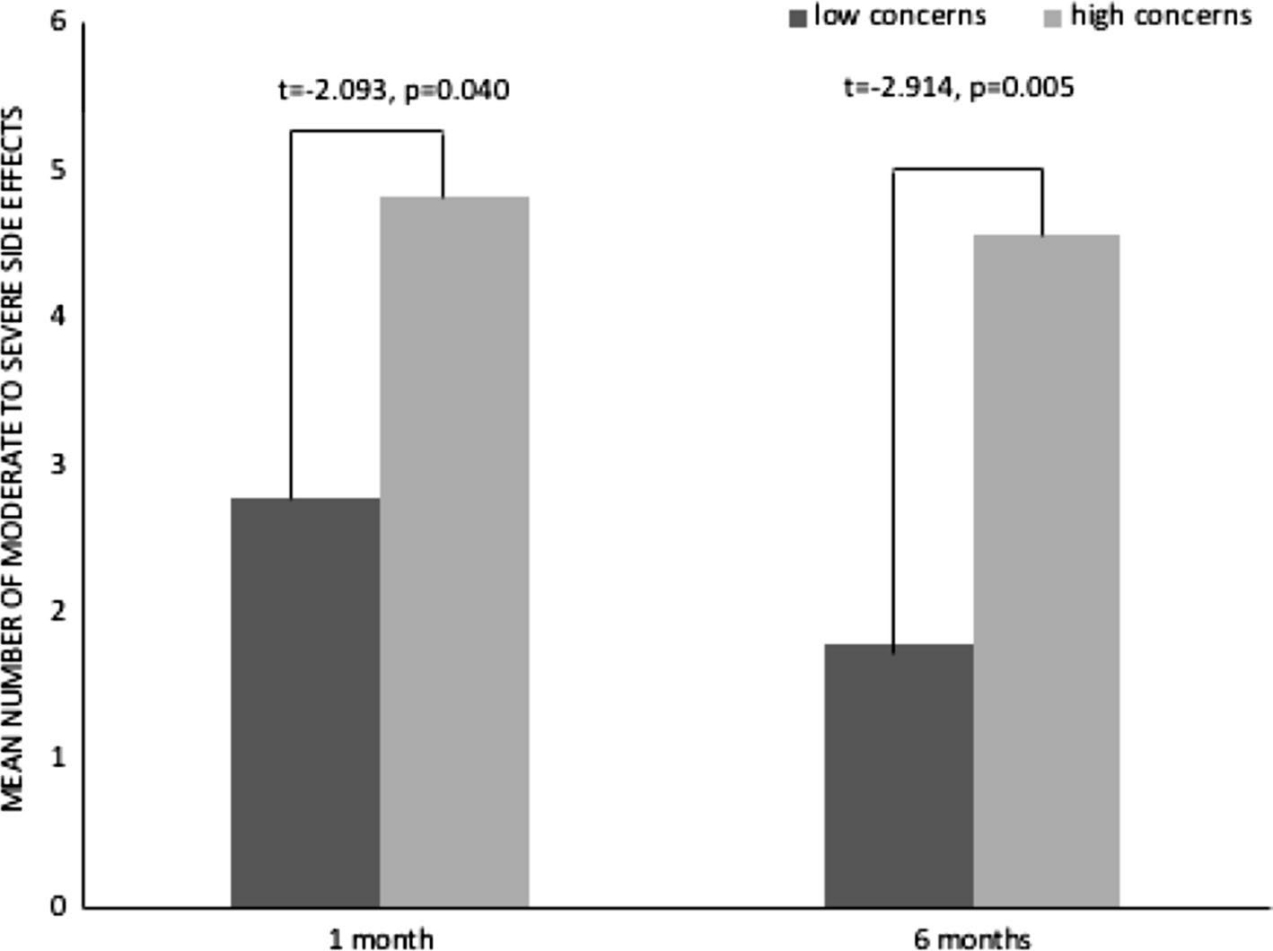
Mean number of moderate, severe or very severe side effects reported at 1 month and 6 months by patients with high concerns and those with low concerns at baseline

**Fig. 2:**
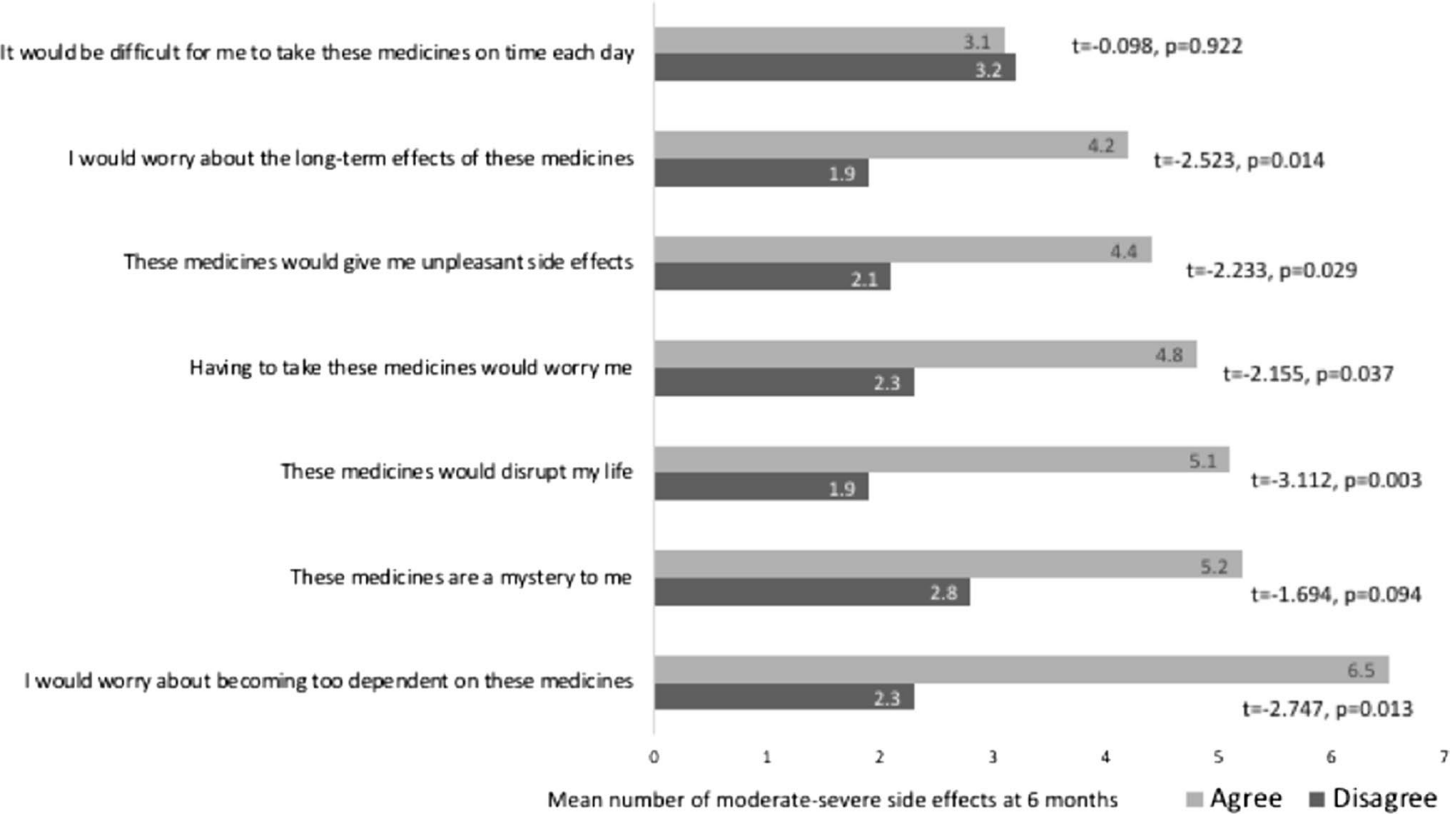
Mean number of moderate, severe or very severe side effects reported at 6 months by patients endorsing each of the BMQ ART-Concerns items at baseline

Consistent with expectations, reporting of moderate to severe side effects at 1 month and 6 months was associated with BMQ ART-Concerns at baseline (Table 3; Fig. 1). Specific concerns about ART associated with side effects at 6 months are shown in Fig. 2. Side effects at 1 month and 6 months were also strongly associated with depression and anxiety scores on the HADS (Table 3). With the exception of duration of HIV, reporting of side effects at 1 and 6 months was not associated with baseline demographic or clinical variables (age, previous ART prescription, diagnosis of AIDS, CD4 count, viral load log_10_; Table 3).

**Table 3:** Baseline predictors of side effects at 1 month and 6 months

		1 month		6 months	
		r or r_p_	p	r or r_p_	p
Age	r	− 0.012	0.916	0.038	0.744
White British	r_p_	− 0.112	0.334	− 0.097	0.405
Men who have sex with men (MSM)	r_p_	0.201	0.081	0.114	0.326
Employed	r_p_	− 0.197	0.088	− 0.184	0.112
AIDS diagnosis	r_p_	− 0.021	0.859	− 0.104	0.369
Prior antiretroviral therapy (ART)	r_p_	0.216	0.061	0.114	0.325
Protease-inhibitor-based ART regimen	r_p_	− 0.722	0.474	− 0.013	0.990
CD4 count (mm^3^/L)	r	0.151	0.193	0.108	0.353
Viral load (log_10_ copies/mL)	r	0.103	0.378	0.144	0.216
Duration of HIV diagnosis (years)	r	0.281	0.014	0.296	0.010
BMQ ART-Necessity	r	− 0.177	0.125	− 0.203	0.079
BMQ ART-Concerns	r	0.347	0.002	0.360	0.001
BMQ ART-General Overuse	r	− 0.113	0.383	− 0.103	0.425
BMQ ART-General Harm	r	0.201	0.116	0.178	0.166
BMQ ART-General Benefit	r	− 0.147	0.259	− 0.144	0.267
Perceived Sensitivity to Medicines (PSM)	r	0.262	0.045	0.241	0.066
HADS Depression	r	0.403	0.000	0.450	< 0.001
HADS Anxiety	r	0.454	0.000	0.440	< 0.001

*BMQ* beliefs about medicines in general, *HADS* hospital anxiety and depression scale

### Determinants of ART Concerns

ART-Concerns at baseline were significantly associated with BMQ General-Harm beliefs, perceived sensitivity to medicines, and symptoms of depression and anxiety (Table 4). ART-Concerns at baseline were not significantly associated with baseline demographic characteristics, previous experience of ART or any other clinical variable (Table 4).

**Table 4:** Associations between baseline demographic, clinical and psychological variables and ART Concerns at baseline

		*r* or *r*_*p*_	*p*
Age	r	− 0.186	0.108
White British	r_p_	0.024	0.834
Men who have sex with men (MSM)	r_p_	0.067	0.563
Employed	r_p_	− 0.045	0.701
AIDS diagnosis	r_p_	− 0.062	0.592
Prior antiretroviral therapy (ART)	r_p_	0.088	0.447
Protease-inhibitor based ART regimen	r_p_	− 0.077	0.508
CD4 count (mm^3^/L)	r	0.148	0.202
Viral load (log_10_ copies/mL)	r	0.088	0.450
Duration of HIV diagnosis (years)	r	0.101	0.386
Beliefs about medicines questionnaire—general overuse	r	0.054	0.677
Beliefs about medicines questionnaire-general harm	r	0.298	0.019
Beliefs about medicines questionnaire—general benefits	r	− 0.162	0.211
Perceived sensitivity to medicines	r	0.545	< 0.001
Hospital anxiety and depression scale—depression	r	0.536	< 0.001
Hospital anxiety and depression scale—anxiety	r	0.547	< 0.001

### Mediational analyses

In line with our hypothesis, BMQ ART-Concerns at baseline was correlated with the outcome, side effects at 6 months (r = 0.360; p < 0.01). Also, BMQ ART-Concerns at baseline was significantly correlated with the proposed mediators. anxiety and depression at 1 month (anxiety: r = 0.506; p < 0.0001; depression: r = 0.5318; p < 0.0001). Furthermore, anxiety and depression at 1 month were correlated with side effects at 6 months (anxiety: r = 0.501; p < 0.0001; depression: r = 0.542; p < 0.0001). The mediation effect induced by anxiety and depression at 1 month was demonstrated through the construction of a model using linear regression equations where anxiety and depression at 1 month were respectively taken as a criterion variable and ART concerns at baseline as a predictor, and a multilinear regression equation taking side effects at 6 months as the criterion variable and BMQ ART-Concerns at baseline and anxiety and depression at 1 month as predictors (F = 10.850; df = 3; p < 0.001). Applying Baron and Kenny’s equation [32] to assess whether depression and anxiety at 1 month mediate the relationship between the causal variable (ART concerns at baseline), and the outcome (side effects at 6 months), resulted in ratio of 0.78. Since the size of the indirect effect was < 0.80 [32], this suggests that anxiety and depression at 1 month partially mediate the relationship between baseline ART-Concerns and side effects at 6 months.

## Discussion

This is the first study to demonstrate that beliefs about medicines predict subsequent reporting of ART side-effects. Patients’ concerns about ART at the time of the treatment recommendation influenced their subsequent experience of side effects. Side effects were associated with subsequent non-adherence.

A previous analysis of data from this study showed that patients’ beliefs about ART at treatment offer were strong predictors of subsequent non-adherence to ART. Specifically, patients who had strong concerns about ART and those who had doubts about their personal necessity for ART were more likely to be non-adherent during the subsequent year [12]. The findings of the current analysis show that medication beliefs also influence patients’ experiences of symptoms attributed to medication side effects.

Our findings are consistent with those of previous studies in other long-term conditions, which found that patients who had negative expectations of treatment or stronger concerns about their medication were more likely to experience side effects [15, 16, 19]. To our knowledge only one previous study has examined the relationship between treatment concerns and side effect experiences in people living with HIV [14]. Gonzalez et al. found a significant association between medication beliefs and symptom reports, but were unable to determine the direction of the relationship due to the cross-sectional study design [14]. Through the use of a prospective, longitudinal design, the current study was able to ascertain that patients’ concerns about medicines before they started treatment predicted the subsequent reporting of ART side effects.

The association between depression and anxiety and experience of side effects is consistent with the findings of prospective studies of patients taking treatment for cancer and with people undergoing vaccination [16, 20]. The results of the mediational analysis conducted in the current study suggests that initiating treatment with strong concerns about ART may lead to depression and anxiety which in turn may impact on the experience of side effects. Further research, using a randomised controlled trial, will determine whether addressing ART concerns at baseline can reduce subsequent ART side effects, and whether depression and anxiety mediate this effect.

Consistent with studies in other long-term conditions we found concerns about ART were influenced by patients’ beliefs about medicines in general, specifically beliefs that medicines are fundamentally harmful, addictive substances that should not be taken for long periods of time [21] and perceptions of personal sensitivity to adverse effects [21]. Other factors, such as the degree of trust that the patient has in their healthcare team have also been found to influence patients’ concerns about ART but were not measured in this study [13].

Limitations of the study included a low completion rate due to people stopping treatment, failing to return questionnaires and missing data in returned questionnaires. Those with missing data were younger, more likely to have been previously prescribed ART and had more negative beliefs about medicines and higher depression and anxiety scores than those who provided complete data, all of which have been associated with low adherence [12]. The study sample may therefore over-represent patients who are highly adherent to their treatment. Data were collected between 2000 and 2004, since which time ART regimens have changed and are considerably better tolerated, therefore people taking ART are likely to report fewer side effects. Since 2004, there have also been changes to treatment guidelines [22], meaning that many people now start treatment at a much higher CD4 count than at the time of the study, and therefore may report fewer symptoms overall. As result of missing data, the study lacked power for multivariate statistics. It was therefore not possible to determine the relative effect of beliefs about medicines and other variables on the experience of side effects. Furthermore, the study was not sufficiently powered to test the impact of the different ART regimens on side effect reports. It is likely that the profile of side effects reported by patients was affected by the medication they were taking and whether they changed medication over the course of the study. Larger datasets, allowing for the comparison of HIV side effect reports in different patient groups, would be needed to test whether the associations between beliefs, symptom reports and adherence seen within this study are consistent across different medication regimens. Multiple comparisons were made, increasing the possibility that one or more significant findings were due to chance (Type 1 error). We relied on self-report measures, including a self-report measure of adherence, which may be subject to a positive bias.

## Conclusion

This study highlights the importance of patients’ pretreatment beliefs about ART as determinants of their subsequent experience of side effects and nonadherence. Our findings are relevant to clinical practice and the design of interventions to improve patients’ experiences of ART and promote adherence. They suggest that exploring and addressing patients’ concerns about ART before they initiate ART may prevent nonspecific side effects and improve patients’ experience of treatment. This, together with the continual development of new drugs that have fewer side effects, may improve patients’ experiences of ART and thereby facilitate adherence.
